# Alternative normalization and analysis pipeline to address systematic bias in NanoString GeoMx Digital Spatial Profiling data

**DOI:** 10.1016/j.isci.2022.105760

**Published:** 2022-12-09

**Authors:** Levi van Hijfte, Marjolein Geurts, Wies R. Vallentgoed, Paul H.C. Eilers, Peter A.E. Sillevis Smitt, Reno Debets, Pim J. French

**Affiliations:** 1 Department of Neurology, Brain Tumor Center at Erasmus MC Cancer Center, 3015 GD Rotterdam, the Netherlands; 2 Laboratory of Tumor Immunology, Department of Medical Oncology, Erasmus MC University Medical Center, 3015 GD Rotterdam, the Netherlands; 3 Department of Biostatistics, Erasmus MC University Medical Center, 3015 GD Rotterdam, the Netherlands

**Keywords:** Biocomputational method, Data processing in systems biology, Transcriptomics

## Abstract

Spatial transcriptomics is a novel technique that provides RNA-expression data with tissue-contextual annotations. Quality assessments of such techniques using end-user generated data are often lacking. Here, we evaluated data from the NanoString GeoMx Digital Spatial Profiling (DSP) platform and standard processing pipelines. We queried 72 ROIs from 12 glioma samples, performed replicate experiments of eight samples for validation, and evaluated five external datasets. The data consistently showed vastly different signal intensities between samples and experimental conditions that resulted in biased analysis. We evaluated the performance of alternative normalization strategies and show that quantile normalization can adequately address the technical issues related to the differences in data distributions. Compared to bulk RNA sequencing, NanoString DSP data show a limited dynamic range which underestimates differences between conditions. Weighted gene co-expression network analysis allowed extraction of gene signatures associated with tissue phenotypes from ROI annotations. Nanostring GeoMx DSP data therefore require alternative normalization methods and analysis pipelines.

## Highlights

• NanoString GeoMx DSP data are hindered by a systematic technical bias
• Data distributions are incompatible with standard analysis due to background noise
• Quantile normalization corrects technical bias and restores biological signal
• Substitute analysis identifies biologically meaningful spatial gene signatures

## Graphical abstract

**Figure:**
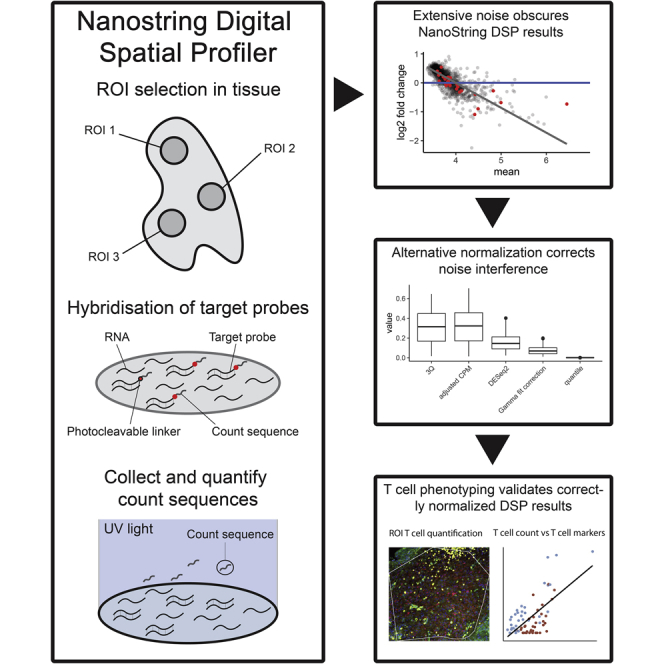

## Introduction

Tissue heterogeneity is a key feature of cancer that likely underlies treatment resistance.^1^ In glioma, heterogeneity is already apparent microscopically when assessing glioma on glioma H&E sections and extends to the molecular level with different regions of the tumor harboring distinct genomic and transcriptomic aberrations.^1^ In addition to the heterogeneity regarding neoplastic cells, various regions of glioma also show differential abundances of tumor cells and other cell populations (e.g. glial cells, neurons, myeloid cells, and endothelial cells).^1^^,^^2^^,^^3^ Single-cell RNA sequencing further showed the extensive diversity in expression profiles of both tumor cells and infiltrating myeloid cells, and pointed toward the relevance of interactions between malignant and non-malignant cells for tumor evolution and therapy response.^4^^,^^5^

To better understand the heterogeneity of malignancies like glioma, spatially resolved transcriptomics techniques have been developed.^6^ To enable readouts specifically for formalin-fixed and paraffin-embedded (FFPE) tissue sections, NanoString developed the GeoMx digital spatial profiler (DSP) that allows collection of RNA expression data from manually selected regions of interest (ROIs). To this end, DSP relies on *in situ* hybridization probes that are linked to a UV cleavable barcode for selective local readouts. This platform is an attractive and user-friendly option as NanoString provides a standard analysis platform that integrates all the required bioinformatical (preprocessing) tools.

The emergence of spatially resolved transcriptomics provides opportunities as well as challenges.^7^ A fundamental challenge regarding the accurate analysis of transcriptomics data is appropriate normalization with the intent to limit the impact of technical variability. The DSP platform is widely used to study spatial heterogeneity of gene expression, yet the quality and validity of the data, including its normalization and downstream analysis, have not been rigorously assessed. Here, using the DSP platform, we have systematically evaluated data from a set of paired isocitrate dehydrogenase-1 (IDH1) R132H mutant glioma samples. We demonstrate that after normalization according to standard strategies, data obtained from this platform do not conform to basic assumptions underlying RNA-seq analysis and produce a heavy bias which leads to incorrect biological interpretation of results. Compared to bulk RNA sequencing, the data show a decreased range in expression levels which limits the ability of the Nanostring DSP platform to detect differences between conditions and underestimates the level of any difference. We propose a renewed and validated processing workflow that addresses the technical bias and skewed data obtained with this platform.

## Results

### Large differences in data distributions between study populations make data incompatible with downstream analysis

In this study, we have assessed the quality of data from the NanoString GeoMx DSP platform for 12 resections obtained from six patients with IDH1-R132H mutant glioma (patient characteristics are listed in Table 1). All patients underwent two resections of which all first resections were low-grade astrocytoma (WHO 2016 grade II or III^8^) and second resections were glioblastoma (WHO 2016 grade IV). Within FFPE sections, tumor-rich ROIs were selected that either had a high T cell number (up to 94 T cells per region) or no T cells according to immunofluorescent tagging of tumor cells (IDH1-R132H), leukocytes (CD45), and T cells (CD3). ROI selection on the NanoString GeoMx DSP is based on direct fluorescent labeling of the primary antibody. We therefore confirmed staining accuracy with standard secondary antibody stainings of consecutive tissue sections (Figure S1). The NanoString Cancer Transcriptome Atlas (CTA) panel was used for the GeoMx DSP experiments, which consists of ∼1800 genes that were selected for their involvement in tumor development and immune response.

**Figure 1:**
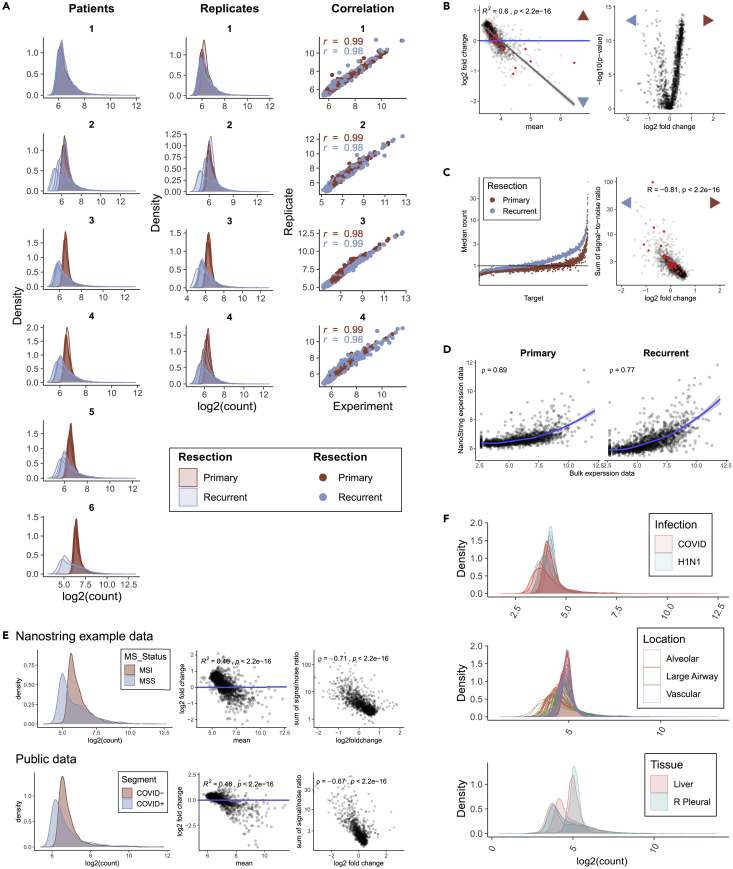
Quality control of NanoString GeoMx DSP data reveals systematic differences in signal-to-noise ratio between study populations (A) Data distributions of all ROIs after Q3 normalization. Left: 12 tumor samples of 6 patients showing large difference in signal distribution between primary and recurrent resections as well as between patients. Middle: replicate experiments for 8 samples of 4 patients. Right: Comparison of gene expression between replicate experiments. Pearson correlation between replicates is shown. (B) Comparison of primary and recurrent samples for Q3-normalized data. Left: MA plot comparing mean expression data between primary and recurrent samples. Regression line is shown in gray with SE. Right: volcano plot showing differential gene expression thereof. Red dots indicate housekeeping genes. (C) The signal-to-noise ratio differs between primary and recurrent samples. Left: scatterplot of ranked median signal-to-noise ratio for all RNA targets per resection type. Right: Scatterplot comparing LFC with the sum of the median signal-to-noise ratio from primary and recurrent samples. Red and blue arrowheads indicate upregulated gene expression for primary and recurrent samples, respectively (data of individual samples shown in Figure S2). (D) Scatterplots comparing NanoString GeoMx DSP and bulk RNA expression data for primary (left) and recurrent (right) samples. Rho indicates Spearman’s rank-order correlation. Loess regression line is shown in blue with SE in gray. (E) Difference in signal-to-noise ratio interferes with data from external datasets. Top: NanoString example data (MSI: microsatellite instable; MSS: microsatellite stable). Bottom: Publicly available dataset. Left: Density plots of grouped data as defined by the experimental design. Middle: MA plots of Q3-normalized data comparing groups as defined in the density plots. Right: Scatterplots showing LFC compared to the signal-to-noise ratios. (F) Density plots showing data distributions of individual ROIs for 3 published datasets from the NanoString GeoMx DSP platform. Colors indicate groups as defined in the original study design.

Raw data counts showed marked differences in signal distributions between, and to a lesser extent, within samples. After data filtering and normalization according to standard manufacturer procedures (i.e., third quartile (Q3) normalization), the differences in signal distribution remained and were most pronounced between primary and recurrent tumors (Figures 1A and S2A). These differences were unexpected and absent in matched bulk sequencing data (Figure S2B). To address laboratory variation, we performed replicate experiments on eight paired samples from four patients. Near-identical distributions were observed with a high correlation between biological replicates (Figure 1A, Pearson correlation of >0.98) which shows that the NanoString GeoMx DSP platform yields reproducible results and that sample-intrinsic properties may underlie the differences in signal distribution. The corresponding MA plot (the log2 fold changes (LFC) between primary and recurrent tumors vs log2 of the mean expression per gene) shows a significant deviation from the y = 0 where all genes, including housekeeping genes, follow the same trend (Figure 1B; R^2^ = 0.6; p < 2.2x10-16). These analyses indicate that the large difference in data distributions (i.e. the range and frequency of counts that occur in a sample) between groups is not caused by a biological signal, and that a technical artifact might cause this problem. Consequently, comparison between primary and recurrent tumor samples will likely become biased and produce erroneous results, as exemplified by the highly asymmetric volcano plot (Figure 1B).

**Table 1:** Patient characteristics

Study_ID	Sex	Age primary resection	Age recurrent resection
1	Male	46	50
2	Female	32	33
3	Male	40	46
4	Male	29	33
5	Male	36	46
6	Male	36	41

### The systematic bias between primary and recurrent samples is caused by a difference in signal-to-noise ratio

The NanoString GeoMx DSP platform uses an indirect readout for RNA expression which is burdened with a background signal. Therefore, a set of control probes (probes that have no target) are used to discern signal from background noise. Several (predominantly primary) samples showed a higher average raw count for control probes and a lower average raw count for target probes compared to other (predominantly recurrent) samples, which resulted in significantly different average signal-to-noise ratio when comparing primary and recurrent samples (Figure S2C, Wilcoxon rank-sum test, p = 2.17×10-3). Even though the signal-to-noise ratio showed considerable inter-patient variation, it remained systematically higher in recurrent samples, and individual targets showed a systematic deviation of the signal-to-noise ratio between primary and recurrent samples (Figures S2D and 1C). No sample-related factors were found to explain differences in signal-to-noise ratio, including the time at which tissue was collected (Figure S2E). We tested the effect of the signal-to-noise ratio on downstream analysis by comparing differentially expressed genes between primary and recurrent tumors. This showed that the signal-to-noise ratio was significantly different for genes with a significant LFC (Figure S2F, Wilcoxon rank-sum test, p < 0.0001). In fact, the signal-to-noise ratio largely determined the outcome of differential gene expression analysis (Figure 1C). The signal-to-noise ratio is not accounted for in the standard Q3 normalization strategy, which does not adjust for possible differences in ranges of data distributions. Q3 normalization uses the third quartile values to align all ROI count measurements. Because primary samples generally have a lower signal-to-noise ratio, their count range will be limited compared to recurrent samples. On average, background noise values of samples with a limited range will thus be higher after Q3 normalization and introduce a systematic bias in data analysis (Figure S2G). Comparisons between median values of bulk RNA-seq and NanoString GeoMx DSP data from the same samples did show some concordance, indicating that the data contain a biologically meaningful signal as well (Figure 1D). However, only highly expressed genes in bulk RNA-seq showed an increasing signal in NanoString GeoMx DSP data, and most low-expressed genes showed a signal that remained constant in NanoString GeoMx DSP data irrespective of bulk sequencing result. Of note, the correlation between bulk RNA-seq and NanoString GeoMx DSP data was higher for recurrent samples (Spearman’s rho of 0.69 and 0.77 in primary and recurrent samples, respectively), which is in line with the higher signal-to-noise ratio in recurrent resections.

### Differences in signal-to-noise cause similar interference in external datasets

The dissimilarities in signal distributions visible in our own dataset were also present in all external datasets we have thus far examined.^9^^,^^10^^,^^11^^,^^12^ Raw data were available for two datasets,^9^ which show clear differences in distribution between groups, skewed MA plots, and a clear influence of the signal-to-noise ratio on the LFC (Figure 1E). For three other external datasets, only normalized data were available^10^^,^^11^^,^^12^ which revealed systematic variation between samples and ROIs depending on study subgroups (Figure 1F). It is interesting to note that the most consistent differences in signal-to-noise ratio, even when subtle, were observed for different input tissues (COVID- vs COVID+, MSI vs MSS colorectal cancer, and primary vs recurrent gliomas). The reason for these differences remains unknown but it severely complicates correct data interpretation. Overall, these validations imply that differences in signal-to-noise ratio severely affect outcomes independent of tissue type or sample size.

### Alternative normalization methods correct for the technical bias in the data

**Figure 2:**
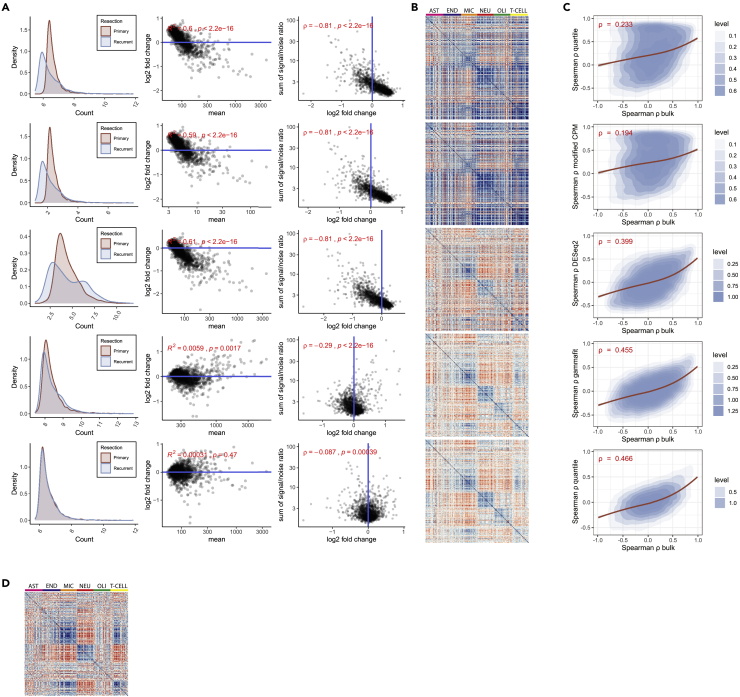
Benchmarking of normalization methods shows that quantile normalization corrects for the bias in NanoString GeoMx DSP data (A) Effect of five normalization approaches on skew in data. From top to bottom: Q3, adjusted CPM, DESeq2, gamma fit correction, and quantile. Left: density plots of grouped data for primary and recurrent resections. Middle: corresponding MA plots comparing primary and recurrent samples. Right: scatterplots comparing sum of signal-to-noise ratio and LFC. Interference of different signal-to-noise ratio remains present in all normalization methods except for quantile normalization. (B) Heatmap showing Spearman correlation for marker genes of major cell types from the CNS. From top to bottom: Q3 normalization (we also performed a linear correction of Q3-normalized data with similarly poor results, data not shown); adjusted CPM; DESeq2 (variance stabilizing transformation); gamma fit correction; and quantile normalization. The scale of blue to red indicates Pearson correlation between 1 and -1. Color bars indicate marker gene clusters for astrocytes (pink), endothelial cells (blue), microglia (orange), neurons (red), oligodendrocytes (green), and T cells (yellow). (C) 2d density plot of Spearman’s rho values for marker genes compared between normalization methods and bulk RNA-seq data. Loess regression line is shown in red. (D) Heatmap showing Spearman’s rho for marker genes of major cell types from the CNS for bulk RNA-sequencing data of corresponding patients (as a reference for corresponding NanoString correlation plots). R^2^ and Spearman’s rho (ρ) are shown in A and C.

As bulk RNA-sequencing data demonstrated that our glioma dataset does contain biologically relevant information (Figure 1E), we considered alternative methods to correct for the differences in signal between samples. To this end, we applied four alternative normalization methods, namely a modified CPM normalization, DESeq2 normalization, gamma fit correction, and quantile normalization. Each method was assessed against three technical criteria in a benchmarking framework to evaluate whether the data are suited for standard differential gene expression testing: (i) Similarity of data distributions between individual samples was tested using the Kolmogorov-Smirnov Test (Figure S2H). (ii) Similarity of average gene expression values between test groups was evaluated by estimating the deviation of the MA plot from y = 0 (Figures 2A and S2I). (iii) Noise interference was estimated using Spearman’s rho for the signal-to-noise ratio as a function of LFC for all targets (Figures 2A and S2I). The tests considered in our benchmarking framework show consistent and significant deviation from assumptions for Q3 normalization. Other methods based on a normalization factor (adjusted CPM, DESeq2) scored comparably bad, while rank-based normalization methods (gamma fit correction and quantile) scored better for all tests (Figures 2A, S2H, and S2I). Quantile normalization yielded the best result for all tests. The rank-based methods evaluated here assume that differences in global variation between the samples do not represent biological data and correct for this. This likely also reduces the influence of signal-to-noise ratio in the data. Indeed, principal component analysis of the samples shows that the contribution of the signal-to-noise ratio in Q3-normalized data highly affects clustering results, while this effect is minimized after quantile normalization (Figure S2J).

In addition to addressing technical criteria in our benchmarking framework, we addressed biological criteria as well. Biological signal was evaluated by testing the correlation of an annotated set of marker genes for the major cell types from the CNS.^13^^,^^14^ Robust biological signal should be reflected in co-expression, and by extension, correlation of marker genes. Virtually no cell-type-specific correlation was observed using the standard Q3 normalization. However, several alternative normalization methods do show correlation between cell-type-specific genes and are similar to correlation patterns from matched bulk RNA-seq samples (Figures 2B and 2D). We evaluated the agreement of Spearman’s rho values between genes from our marker set using bulk RNA-seq as reference for biological signal (Figure 2C). Several alternative methods (gamma fit correction, quantile, and DESeq2) show an improved correlation, with highest agreement for quantile normalization. Taken together, benchmarking using both technical and biological criteria show that quantile normalization most adequately corrects for technical difficulties and restores biological signal in the NanoString GeoMx DSP data. However, the data need to be thoroughly scrutinized before this can be shown with any reliability.

### WGCNA identifies a T cell signature that is correlated with T cell numbers in ROIs

**Figure 3:**
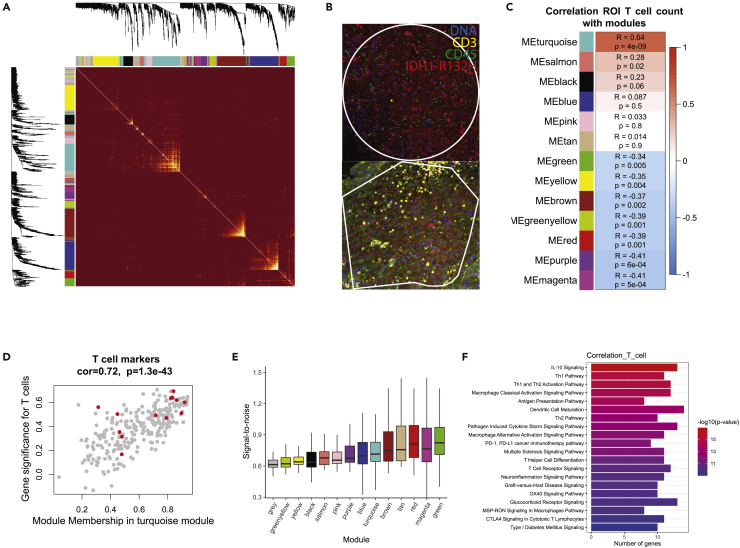
Correlation analysis of ROI T cell count with WGCNA gene modules yields expected outcome from quantile-normalized NanoString data (A) Heatmap showing Topological Overlap Matrix (TOM) scale, which from red to yellow indicates the extent of relative overlap between genes. Dendrogram and gene module colors are depicted along the top and side. (B) Example images of ROIs showing staining for CD3 (yellow), CD45 (green), IDH1-R132H (red), and DNA (blue). (C) Pearson correlation matrix between module eigengenes and ROI T cell count. The scale of red to blue depicts correlation or inverse correlation with ROI T cell count, respectively. (D) Scatterplot for genes of the turquoise module showing correlation between the turquoise Module Membership scores and p values for the association of genes with ROI T cell count. Red dots indicate T cell marker genes from an annotated gene set.^14^ (E) Boxplot showing signal-to-noise ratio for the genes in all clusters. (F) IPA analysis for the top 50 genes from the turquoise module. All boxplot boxes show the interquartile range (IQR) whiskers represent 1.5 x IQR.

To test whether we can extract information on T cell biology, we used weighted correlation network analysis (WGCNA) on the quantile-normalized data.^15^ WGCNA subdivided the 1673 genes into thirteen modules on the basis of mutual correlations between expression levels (Figure 3A). We subsequently quantified the number of T cells present in the NanoString IF staining of ROIs (Figures 3B and S3A), and tested whether any of the identified modules correlated with T cell count (Figure 3C). The turquoise module was highly correlated with T cells and individual T cell marker genes showed a consistently high score for both the turquoise module and T cell count (Pearson’s R = 0.64, p = 4×10-9, Figures 3C and 3D). WGCNA assigns genes to the gray module if they show no clear correlation pattern in the data. As expected, this module has the lowest signal-to-noise ratio compared to all other modules (Figure 3E). Subsequently, Ingenuity Pathway Analysis (IPA) was performed using the 50 top-ranked genes from the best correlated (turquoise) module. The genes that are correlated with T cell number are involved in general immune activity, particularly including antigen presentation, CD4 T cell activation, and T cell receptor signaling (Figure 3F). Gene modules that are inversely correlated with T cell number show no clear gene set enrichment, likely due to limited power of our reference gene set. By comparison, WGCNA analysis of Q3-normalized data showed unevenly divided modules, effectively identifying one large module. This module showed the lowest average signal-to-noise ratio, implying that it is the consequence of the difference in signal-to-noise between samples in the dataset (Figures S3B and S3C). These data demonstrate that biological signals can be discerned from the NanoString GeoMx DSP platform, but only when using normalization methods and downstream analysis that take the limitations of this technique and the data into account.

## Discussion

The NanoString GeoMx DSP platform provides spatially resolved expression data coupled with easy-to-use software with build-in preprocessing tools. This allows users to focus directly on the biological interpretation of data. While convenient, it remains imperative to assess whether underlying data properties are appropriate for downstream analysis to substantiate the validity of the conclusions drawn from it.

Comparative transcriptomics analysis generally assumes that most genes are not differentially expressed between conditions and thus follow a similar data distribution. In this study, we show that after Q3 normalization this assumption does not hold for any of the datasets that were evaluated, and that the data are heavily skewed. To address this issue, we evaluated the performance of four recognized normalization methods. We show that these methods uphold basic assumptions better compared to standard Q3 normalization. Of the normalization methods that were tested, quantile normalization was superior across our benchmarking parameters and yielded the required data distributions, providing the most comparable biological results with bulk RNA seq data.

Q3 and quantile normalization are radically different in their approach. Q3 normalization relies a normalization factor that aligns the third quartile gene count value for all samples. This method does not address potential global variations in data distributions or expression ranges. In contrast, quantile normalization, which is widely used to normalize gene expression data derived from microarrays, aligns count values according to the rank of the genes and forces the data of all samples into the same distribution. It is therefore inherent to this method that the difference in count value range or signal-to-noise ratio is no longer dependent on raw data distributions.

The underlying cause for the difference in data distribution seems to be rooted in how well the biological signal exceeds the noise signal. We were not able to find any consistencies in features like tissue age or processing methods that could explain the different data distributions. As primary and recurrent samples for each patient were processed together on one microscope slide, we judged the chance of batch effects to be limited. Possibly, the differences in signal-to-noise ratio are linked to tissue-type-specific qualities that make it more or less suitable for a readout using the NanoString GeoMx DSP platform.

In summary, we demonstrate the critical requirement to assess the correctness of data distribution, particularly regarding the use of the NanoString GeoMx DSP platform. The high level of inter-sample variation can, at least partially, be overcome with alternative normalization strategies. The alternative preprocessing methods and downstream analysis using WGCNA that we present here can provide a starting point for a renewed workflow to correctly interpret the NanoString GeoMx DSP data.

### Limitations of the study

Some limitations of this study should be noted. First, we used bulk RNA-seq to compare with our spatial gene expression data. Even though averaged measurements from the ROIs distributed over a tumor section likely give a balanced pseudo bulk gene expression level, the NanoString GeoMx DSP and bulk RNA-seq methodologies for acquiring count data are very different and should be considered when interpreting the data. Second, it should be noted that we did not exhaustively examine existing normalization methods. The aim of our current study was to illustrate some of the difficulties that novel techniques like the NanoString GeoMx platform face and provide an alternative processing method that helps to overcome them. Third, we do note that quantile normalization, due to its course method of normalization, may limit the detection of more subtle differences in gene expression. Last, the CTA panel measures the expression of a relatively small number of genes (around 1800). We cannot exclude the possibility that the limited panel size has some effect on our test results.

## STAR★Methods

### Key resources table

**Table:** 

REAGENT or RESOURCE	SOURCE	IDENTIFIER
**Antibodies**		

Rabbit monoclonal anti-CD3	Ventana Medical Systems	clone 2GV6, cat#790-4341, RRID:AB_2335978
Rabbit monoclonal anti-CD4	Ventana Medical Systems	clone PS35, cat#790-4423, RRID:AB_2335982
Rabbit monoclonal anti-CD8	Ventana Medical Systems	clone SP57, cat#790-4460, RRID:AB_2335985
Mouse monoclonal anti-IDH1-R132H	Dianova	clone H09, cat#DIA-H09, RRID:AB_2335716

**Biological samples**		

FFPE human glioma samples	Erasmus Medical Center	www.erasmusmc.nl

**Chemicals, peptides, and recombinant proteins**		

CC1	Ventana Medical Systems	cat#950-500
Ultramap anti-rabbit HRP	Ventana Medical Systems	cat#760-4315
CC2	Ventana Medical Systems	cat#950-123
Red610	Ventana Medical Systems	cat#760-245
FAM	Ventana Medical Systems	cat#760-235
Anti-fading medium	Dako	Cat#S3023

**Critical commercial assays**		

ultraView Universal Alkaline Phosphatase Red Detection Kit	Roche	cat#950-500
RNeasy FFPE kit	Qiagen	cat#73504
Ventana Benchmark Discovery	Ventana Medical Systems	Cat#05987750001

**Deposited data**		

NanoString GeoMx DSP data	This paper	https://doi.org/10.5281/zenodo.7348326

**Software and algorithms**		

R version 4.1.0 (2021-05-18)	R-project^17^	https://www.R-project.org/
TME-Analyzer software	Rijnders et al.^16^	NA
Qiagen Ingenuity Pathway Analysis	Qiagen	https://digitalinsights.qiagen.com/products-overview/discovery-insights-portfolio/analysis-and-visualization/qiagen-ipa/
Adobe Illustrator	Adobe	https://www.adobe.com/products/illustrator.html

**Other**		

QC and normalization methods for NanoString GeoMx DSP data	This paper/GitHub	https://github.com/LevivanHijfte/NanoString_normalization_methods

### Resource availability

#### Lead contact

Requests for resources and additional questions should be directed to Levi van Hijfte (l.vanhijfte@erasmusmc.nl).

#### Materials availability

This study did not generate new unique reagents.

### Experimental model and subject details

Patient information can be found in Table 1. The study was approved by the institutional review board of the Erasmus MC, and all patients provided written informed consent.

### Method details

#### Tissue collection and processing

From the operating theater at the Erasmus Medical Center, twelve IDH mutant glioma tissue samples were collected of six patients who underwent two resections (first resection of low-grade (WHO 2016 grade II/III) astrocytoma, second resection of glioblastoma (WHO 2016 grade IV)). Immediately after resection, tissue was fixed in formalin and embedded in paraffin according to standard pathology procedures. FFPE tissue was cut into five micrometer sections and placed on Apex BOND slides (Leica Biosystems, Wetzlar, Germany, #3800040). Paired tumor samples were placed on the same slide. For all samples, six consecutive slides were prepared for replicate experiments and immune stainings (see below). Slides were shipped to NanoString Seattle for ROI selection, data collection and preprocessing. ROI selection was performed based on an immune staining using four markers (DNA, CD3, CD45, IDH-R132H). A total of 12 ROIs per slide (i.e., ∼six per tumor) were selected. For four of six tumor pairs, replicate experiments were performed. Samples from the primary and replicate experiment were processed together in one batch.

#### Immune stainings

Standardized IF and IHC stainings were performed for CD3 (Ventana Medical Systems, Tucsen, AZ, USA; clone 2GV6; concentration 0.4 μg/mL), CD4 (Ventana Medical Systems; clone PS35; concentration 2.5 μg/mL), CD8 (Ventana Medical Systems; clone SP57; concentration 0.4 μg/mL) and IDH1-R132H (Dianova, Hamburg, Germany; clone H09; dilution 1/800). For CD3, CD4 and CD8 a multiplex was performed using the automated Ventana Benchmark Discovery (Ventana Medical Systems). First, antigen retrieval was performed using CC1 (Ventana Medical Systems, cat. no. 950-500) for 64 min at 97°C. Subsequently, samples were incubated with CD3 antibody at 37°C for 64 min, after which antibody detection was performed for 12 min with Ultramap anti-rabbit HRP (Ventana Medical Systems, cat. no. 760–4315), and visualization was performed for 8 min with R6G (Ventana Medical Systems, cat. no. 760-243). CC2 (Ventana Medical Systems, cat. no. 950-123) was used for denaturation at 100°C for 20 min. Second, samples were incubated with the CD4 antibody at 37°C for 32 min followed by antibody detection (as described above) and visualization for 4 min with Red 610 (Ventana Medical Systems, cat. no. 760-245). Denaturation was again performed with CC2 (as described above). Third, slides were incubated with CD8 antibody for 32 min at 37°C followed by antibody detection (as described above) and visualization for 12 min with FAM (Ventana Medical Systems, cat. no. 760-235). Last, slides were incubated in PBS with DAPI for 15 min and mounted with anti-fading medium (Dako, Glostrup, Denmark, cat. no. S3023).

For IDH1-R132H, a single staining was performed on a Ventana Benchmark ULTRA (Ventana Medical Systems, Tucsen, AZ, USA) using the Ultraview universal alkaline phosphatase red detection kit (Roche, Bazel, Switzerland cat. no. 760-501). After deparaffinization and heat-induced epitope retrieval, Incubation of the primary antibody was performed for 32 min at 37°C. Counterstain was performed with hematoxylin II for 12 min and with a bluing reagent for 8 min.

#### NanoString GeoMx DSP data processing and analysis

Quantification of the number of CD3 positive cells in the selected ROIs was done using the TME-Analyzer software (version 2.3).^16^ All data analysis was performed in R.^17^ All visualizations were made using the ggplot2 and ComplexHeatmap R packages. Quality control and filtering was done according to manufacturer’s protocols (MAN-10119-01 GeoMx-NGS Data Analysis User Manual). 8684 probes (SpikeIn probes included) were used to measure expression of 1825 targets (3–5 probes per target). Probes were excluded from analysis if counts were too low (e.g. not exceeding 5 reads in more than 2 ROIs), if probes failed the global outlier test (e.g. the ratio of the geometric mean for individual probes over all ROIs to the geometric mean of individual targets over all ROIs should not be below 0.1) or if probes failed the Grubbs outlier test (e.g. probes should not consistently (in more than 20% of ROIs) be a Grubbs outlier between probes for the same target in one ROI). In total, 135 probes were excluded. Three ROIs were excluded from further analysis because the geometric mean for the Spike-In probe count was below 10. Replicate experiments did not meet one QC requirement due to probe contamination (interference of count probes belonging to a NanoString panel distinct from the CTA panel used for these experiments). However, this did not affect hybridization of CTA panel probes as the number of probes that could be mapped back to the CTA panel reference was satisfactory for all ROIs, and no other QC measures were insufficient. Four ROIs were excluded because the geometric mean for the Spike-In probe count was below 10 and 185 probes were excluded because they failed one of the QC measures described above. After data filtering, the geometric mean was calculated for targets in each ROI. Results for QC were compared to the standard NanoString pipeline results, which overall showed similar results in detection of outliers, with small differences probably due to subtle differences in test parameters. Next, the limit of quantification (LOQ) was calculated for each ROI. LOQ was defined as two standard deviations above the geometric mean of the negative control probe counts (88 ERCC spike-in probes were used as negative control probes for each ROI), as is standard in NanoString workflow. Target values were excluded from analysis if no measurement for that target gene exceeded the LOQ. After filtering of targets using the LOQ, 1673 genes remained in the original dataset, and 1689 genes remained in the validation dataset. Several normalization methods were performed. Third quartile (Q3) normalization was performed according to NanoString guidelines. For the modified CPM normalization, counts for one target in one ROI were divided by the sum of all counts in one ROI and multiplied by a scaling factor (in this case 10,000). Gamma fit correction was performed using the fitdist function from the fitdistrplus R package. Quantile normalization was performed using the normalize.quantiles function from the preprocessCore R package. To acquire DESeq2 normalized data, geometric mean count values were rounded and used as input for the DESeq2 package. VST-transformed values were extracted using the vst function. After normalization, data was log2 transformed for all normalization methods except for DESeq2 normalized data. Since no count values were below 1, no pseudo count was added. The log2 fold change (lfc) was defined as follows: lfc(Ac,Bc) = log2(Ac/Bc) where Ac and Bc represent median counts of gene c for conditions A and B. Signal-to-noise ratio was defined as the ratio of geometric mean of target probes to the LOQ in individual ROIs.

#### Bulk RNA sequencing data QC and normalization

For bulk RNA sequencing of the samples identical to those used for NanoString GeoMx DSP were evaluated for high tumor content by a neuropathologist on an HE stained section. 10 μm sections were made, deparaffinized and macro-dissection was performed according to annotated high-tumor regions for RNA extraction for 5-20 sections. RNA was isolated using the RNeasy FFPE kit (Qiagen, Venlo, The Netherlands cat#73504). Whole transcriptome sequencing was performed by GenomeScan (Leiden, The Netherlands) on rRNA depleted cDNA at depth of 50 million paired-end reads per sample. Raw FASTQ files were provided by manufacturer. Reads were trimmed for low quality bases, adapters were trimmed and low quality reads were discarded using fastp:

*fastp -y -x -p --thread 4 -i < …>_R1.fastq.gz -I <...>_R2.fastq.gz -o <...>_trimmed_R1.fastq.gz -O <...>_trimmed_R2.fastq.gz -j <...>_fastp_log.json -h <...>_fastp_log.html --trim_poly_g -l 35*.

These processed FASTQ files were subsequently aligned with STAR 2.7.3a

*STAR --outFileNamePrefix "<...>/" --runThreadN 6 --genomeLoad LoadAndKeep --limitBAMsortRAM 5000000000 --outTmpDir /dev/shm/<...> --genomeDir /mnt/data/ccbc_environment/general/genomes/hsapiens/GRCh38/STAR/34/ --readFilesIn 'input/fastq-clean/<...>_L004_trimmed_R1.fastq.gz,input/fastq-clean/<...>_L003_trimmed_R1.fastq.gz' 'input/fastq-clean/<...>_L004_trimmed_R2.fastq.gz,input/fastq-clean/<...>_L003_trimmed_R2.fastq.gz' --readFilesCommand zcat --outSAMtype BAM SortedByCoordinate*

to GRCh38 and Gencode 34 into BAM files. Duplicate reads in the bam files were marked using sambamba 0.8.0:

*sambamba-0.8.0-linux-amd64-static markdup -t 6 --tmpdir /dev/shm <…>/Aligned.sortedByCoord.out.bam <…>/Aligned.sortedByCoord.out.markduplicate.bam*.

These BAM files were used to count reads in a stranded manner with featureCounts (subread 2.0.1) and Gencode 34:

*subread-2.0.1-Linux-x86_64/bin/featureCounts -a <…>/hsapiens/GRCh38/gencode/v34/gencode.v34.primary_assembly.annotation.gtf -o GLASS.LGG.EMC.RNA.readcounts.deduplicated_s_2.txt --tmpDir /dev/shm -s 2 -T 24 --primary –ignoreDup –C –p <…1>.bam <…2>.bam*

One sample was excluded from the analysis due to low sequencing depth (<10.000 raw counts). Bulk RNA sequencing data was normalized using the DESeq2 package.^18^ After variance stabilizing transformation (VST), counts were used for comparative analysis.

#### Comparison between GeoMx DSP data and bulk RNA-seq

To compare average RNA expression between bulk and NanoString GeoMx DSP data, we calculated the median normalized counts for all ROIs per resection. To test correlations between marker genes for all normalization methods, we selected the top 50 genes that were enriched in astrocytes, oligodendrocytes, microglia, neurons and endothelial cells from an annotated dataset.^13^ For T cells, a separate list of 48 marker genes was used.^14^ One marker gene for oligodendrocytes and one gene for T cells were excluded because they were absent in bulk RNA seq data. Normalized and log2 transformed counts of all ROIs were used for correlation analysis. Spearman’s Rho values were calculated using the cor function from base R and visualized with the corrplot function from the corrplot R package. To compare correlations, rho values were deduplicated with the upper.tri function from base R.

#### Validation datasets

Five independent datasets were used for validation. Example data for the NanoString GeoMx DSP was kindly provided by NanoString, and published data was recovered from online archives.^9^^,^^10^^,^^11^^,^^12^ Two datasets containing raw reads were processed as described above.^9^ For the NanoString dataset, 19 ROIs were excluded because of a low median spike-in count. For the published dataset, 2 ROIs were excluded due to an insufficient read count (<1000), 2 ROIs were excluded due to a low aligned read to raw read ratio (<80%), and 23 ROIs were excluded due to a low median spike-in count. Probe filtering, LOQ calculation and target filtering were performed as described above. For the three datasets containing normalized data, Q3-normalized and log2 transformed counts were used to visualize data distributions according to study populations designated by the respective study designs.

#### Weighted Gene Co-expression Network Analysis

Weighted Gene Co-expression Network Analysis (WGCNA) was performed using the WGCNA R package.^15^ Because one sample was a consistent outlier in gene correlation analysis and had an outlier profile in WGCNA preprocessing, we excluded it from further analysis. Normalized and log2-transformed data was used as input for this analysis. First, a similarity matrix was constructed of the data using the Pearson correlation. Next, the similarity matrix was transformed into a signed weighted adjacency matrix using a soft thresholding power of 16 that was determined by approximation of the scale-free topology criterion. From this, interconnectedness of all genes was assessed using the topological overlap measure. Using hierarchical clustering for the topological overlap matrix, modules were defined with the Dynamic Tree cut algorithm (integrated in the WGCNA package) with a DeepSplit parameter of 3 and a minimum module size of 30. For WGCNA analysis of the Q3 -normalized data a soft thresholding power of 24 was used. Module membership scores for genes were defined as the Pearson correlation of module eigengenes with the normalized gene expression profiles.

### Quantification and statistical analysis

Statistical analyses were performed using R. For differential gene expression analysis, a Wilcoxon rank-sum rank sum test was performed on all targets and p-values were adjusted using Benjamini-Hochberg correction. For DESeq2 normalized data, log2foldchange and adjusted p-values of the Wald test were extracted using the results function from the DESeq2 R package. Similarity between data distributions was tested using the two-sample Kolmogorov-Smirnov test. The prcomp function was used for principle component analysis. Statistical significance for spearman correlation was calculated using the stat_cor function from the ggpubr package in R. The Pearson correlation between the T cell number and the WGCNA eigengenes of modules was calculated using the cor function in R.

For WGCNA analysis, significance of the correlations was determined using the corPvalueStudent function from the WGCNA package in R. Significance of the relationship between gene count and T cell count was defined as the significance of the Pearson correlation between the T cell number per ROI and gene expression profiles as calculated by the corPvalueStudent function from the WGCNA package. For canonical pathway analysis, we used Qiagen Ingenuity Pathway Analysis (IPA) software (version 56367011) with the filtered NanoString CTA gene target list as a reference. Significance was defined as a p value of <0.05. Specifics on tests and visualizations can be found in the Figure legends.

## Data Availability

• Text files of raw data and sample information of the NanoString GeoMx DSP have been deposited at https://zenodo.org/ and are publicly available as of the date of publication. The DOI is listed in the key resources table. • All original code has been deposited on our GitHub repository (https://github.com/LevivanHijfte/NanoString_normalization_methods) and is publicly available. • Any additional information required to reanalyze the data reported in this paper is available from the lead contact upon request.

## References

[1] Sottoriva A., Spiteri I., Piccirillo S.G.M., Touloumis A., Collins V.P., Marioni J.C., Curtis C., Watts C., Tavaré S. (2013). Intratumor heterogeneity in human glioblastoma reflects cancer evolutionary dynamics. Proc. Natl. Acad. Sci. USA.

[2] Comba A., Faisal S.M., Varela M.L., Hollon T., Al-Holou W.N., Umemura Y., Nunez F.J., Motsch S., Castro M.G., Lowenstein P.R. (2021). Uncovering spatiotemporal heterogeneity of high-grade gliomas: from disease biology to therapeutic implications. Front. Oncol..

[3] Becker A.P., Sells B.E., Haque S.J., Chakravarti A. (2021). Tumor heterogeneity in glioblastomas: from light microscopy to molecular pathology. Cancers.

[4] Hara T., Chanoch-Myers R., Mathewson N.D., Myskiw C., Atta L., Bussema L., Eichhorn S.W., Greenwald A.C., Kinker G.S., Rodman C. (2021). Interactions between cancer cells and immune cells drive transitions to mesenchymal-like states in glioblastoma. Cancer Cell.

[5] Neftel C., Laffy J., Filbin M.G., Hara T., Shore M.E., Rahme G.J., Richman A.R., Silverbush D., Shaw M.L., Hebert C.M. (2019). An integrative model of cellular states, plasticity, and genetics for glioblastoma. Cell.

[6] (2021). Method of the Year 2020: spatially resolved transcriptomics. Nat. Methods.

[7] Atta L., Fan J. (2021). Computational challenges and opportunities in spatially resolved transcriptomic data analysis. Nat. Commun..

[8] Louis D.N. (2016).

[9] Delorey T.M., Ziegler C.G.K., Heimberg G., Normand R., Yang Y., Segerstolpe Å., Abbondanza D., Fleming S.J., Subramanian A., Montoro D.T. (2021). COVID-19 tissue atlases reveal SARS-CoV-2 pathology and cellular targets. Nature.

[10] Brady L., Kriner M., Coleman I., Morrissey C., Roudier M., True L.D., Gulati R., Plymate S.R., Zhou Z., Birditt B. (2021). Inter- and intra-tumor heterogeneity of metastatic prostate cancer determined by digital spatial gene expression profiling. Nat. Commun.

[11] Margaroli C., Benson P., Sharma N.S., Madison M.C., Robison S.W., Arora N., Ton K., Liang Y., Zhang L., Patel R.P., Gaggar A. (2021). Spatial mapping of SARS-CoV-2 and H1N1 lung injury identifies differential transcriptional signatures. Cell Rep. Med..

[12] Rendeiro A.F., Ravichandran H., Bram Y., Chandar V., Kim J., Meydan C., Park J., Foox J., Hether T., Warren S. (2021). The spatial landscape of lung pathology during COVID-19 progression. Nature.

[13] McKenzie A.T., Wang M., Hauberg M.E., Fullard J.F., Kozlenkov A., Keenan A., Hurd Y.L., Dracheva S., Casaccia P., Roussos P., Zhang B. (2018). Brain cell type specific gene expression and Co-expression network architectures. Sci. Rep..

[14] Hammerl D., Massink M.P.G., Smid M., van Deurzen C.H.M., Meijers-Heijboer H.E.J., Waisfisz Q., Debets R., Martens J.W.M. (2020). Clonality, antigen recognition, and suppression of CD8(+) T cells differentially affect prognosis of breast cancer subtypes. Clin. Cancer Res..

[15] Langfelder P., Horvath S. (2008). WGCNA: an R package for weighted correlation network analysis. BMC Bioinf..

[16] Rijnders M., Balcioglu H.E., Robbrecht D.G.J., Oostvogels A.A.M., Wijers R., Aarts M.J.B., Hamberg P., van Leenders G., Nakauma-Gonzalez J.A., Voortman J. (2021). Anti-PD-1 efficacy in patients with metastatic urothelial cancer associates with intratumoral juxtaposition of T helper-type 1 and CD8(+) T cells. Clin. Cancer Res..

[17] C.T. R. (2021). The R project for statistical computing. https://www.R-project.org/.

[18] Love M.I., Huber W., Anders S. (2014). Moderated estimation of fold change and dispersion for RNA-seq data with DESeq2. Genome Biol..

