# Supplementary material for: Alternative normalization and analysis pipeline to address systematic bias in NanoString GeoMx Digital Spatial Profiling data
Source: iScience. 2022 Dec 9;26(1):105760. doi: 10.1016/j.isci.2022.105760 (PMC9800292; doi:10.1016/j.isci.2022.105760)
Supplement: Document S1. Figures S1–S3 [file mmc1.pdf]

iScience, Volume *26*

Supplemental information

Alternative normalization and analysis pipeline to address systematic bias in NanoString GeoMx Digital Spatial Profiling data

Levi van Hijfte, Marjolein Geurts, Wies R. Vallentgoed, Paul H.C. Eilers, Peter A.E. Sillevis Smitt, Reno Debets, and Pim J. French

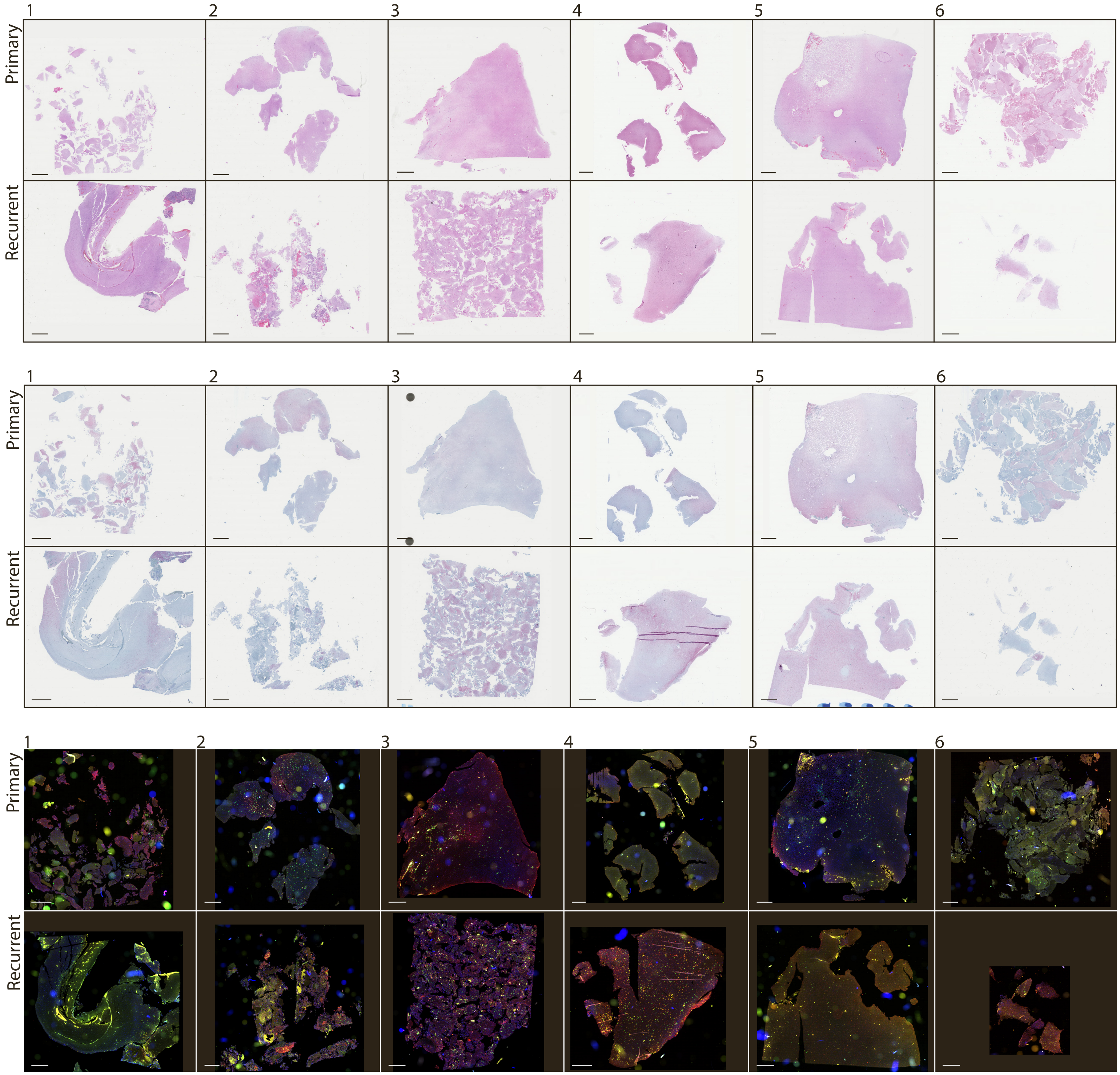

Supplementary Figure 1, related to Figure 1 | Hematoxylin & Eosin (H&E) and Immunohistochemistry (IHC) stainings of consecutive slides of glioma tissues used for NanoString GeoMx DSP. Top: H&E. Middle: IHC staining for IDH1-R132H. Bottom: multiplex immunofluorescence staining for CD3 (yellow), CD4 (green), CD8 (red) and DNA (blue). Scale bars show 2mm.

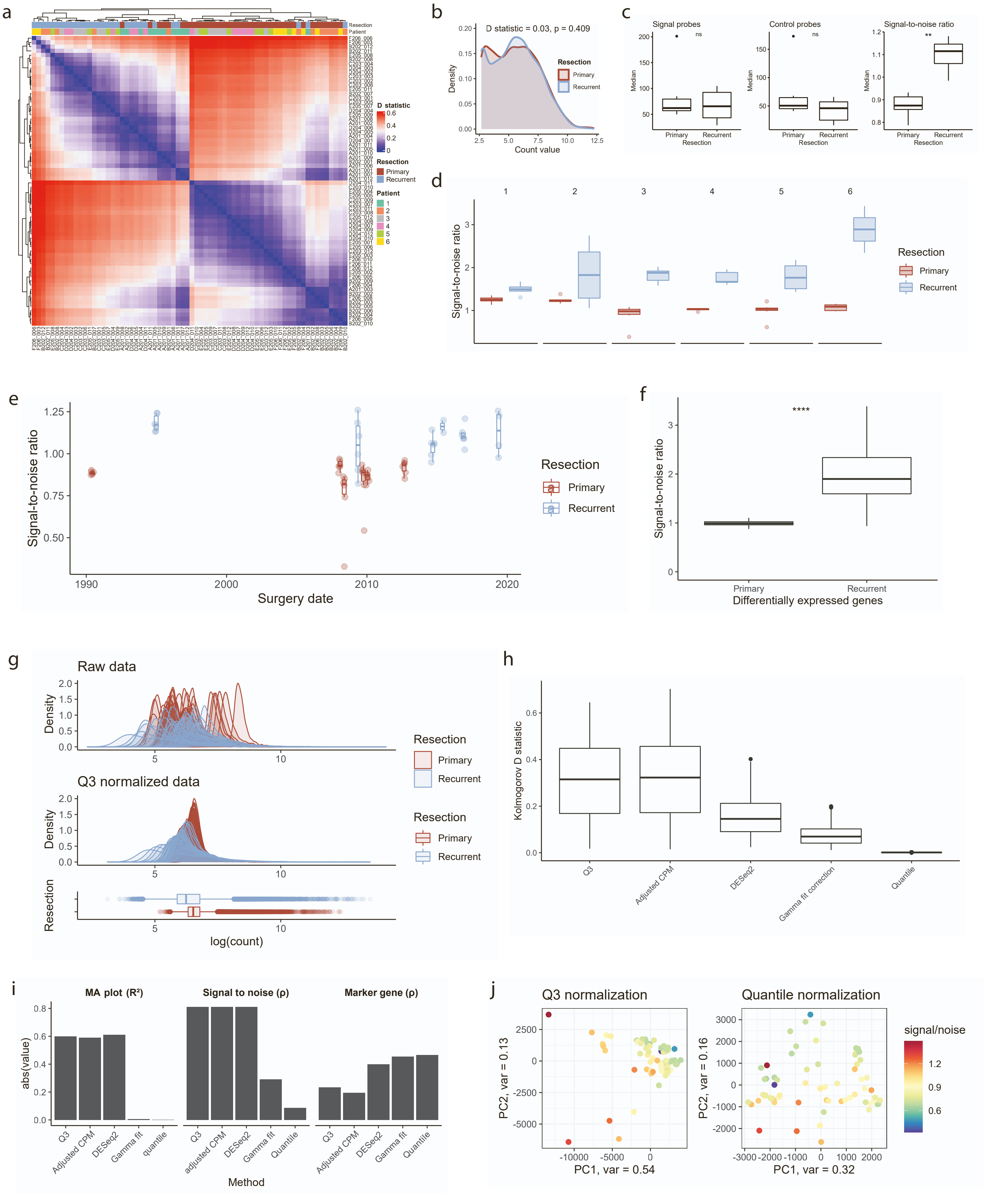

Supplementary Figure 2, related to Figure 1 and Figure 2 | Systematic differences in global data distributions are caused by differences in signal-to-noise ratio in NanoString GeoMx DSP data. (a), Heatmap showing the D statistic from the Kolmogorov-Smirnov test between all data distributions after Q3 normalization. (b), Average data distributions for bulk RNA-seq data. (c), Boxplots of median raw counts per tumour for control probes, target probes and signal-to-noise ratio. (d), Boxplot of average ROI signal-to-noise ratio split for primary and recurrent resections of individual patients. (e), Boxplot of average ROI signal-to-noise ratio per surgery date. (f), Boxplot of signal-to-noise ratio for differentially expressed genes between primary- and recurrent resections. (g), Density plots of raw and Q3 normalized data. Boxplot showing the distribution of all Q3 normalized data. (h), Boxplots showing the range of the Kolmogorov D statistic for comparisons between distributions of all samples in 5 normalization strategies. (i), Barplots of the correlation values from Figure 2a and c. (j), PCA plots of ROIs. Color indicates signal-to-noise ratio. The Wilcoxon rank-sum exact test was used for comparison of groups in c and f. All boxplot boxes show the interquartile range (IQR) whiskers represent 1.5 x IQR. p-values are indicated as follows: ns: not significant, * : $p < 0.05$; * : $p < 0.01$; *** : $p < 0.001$.

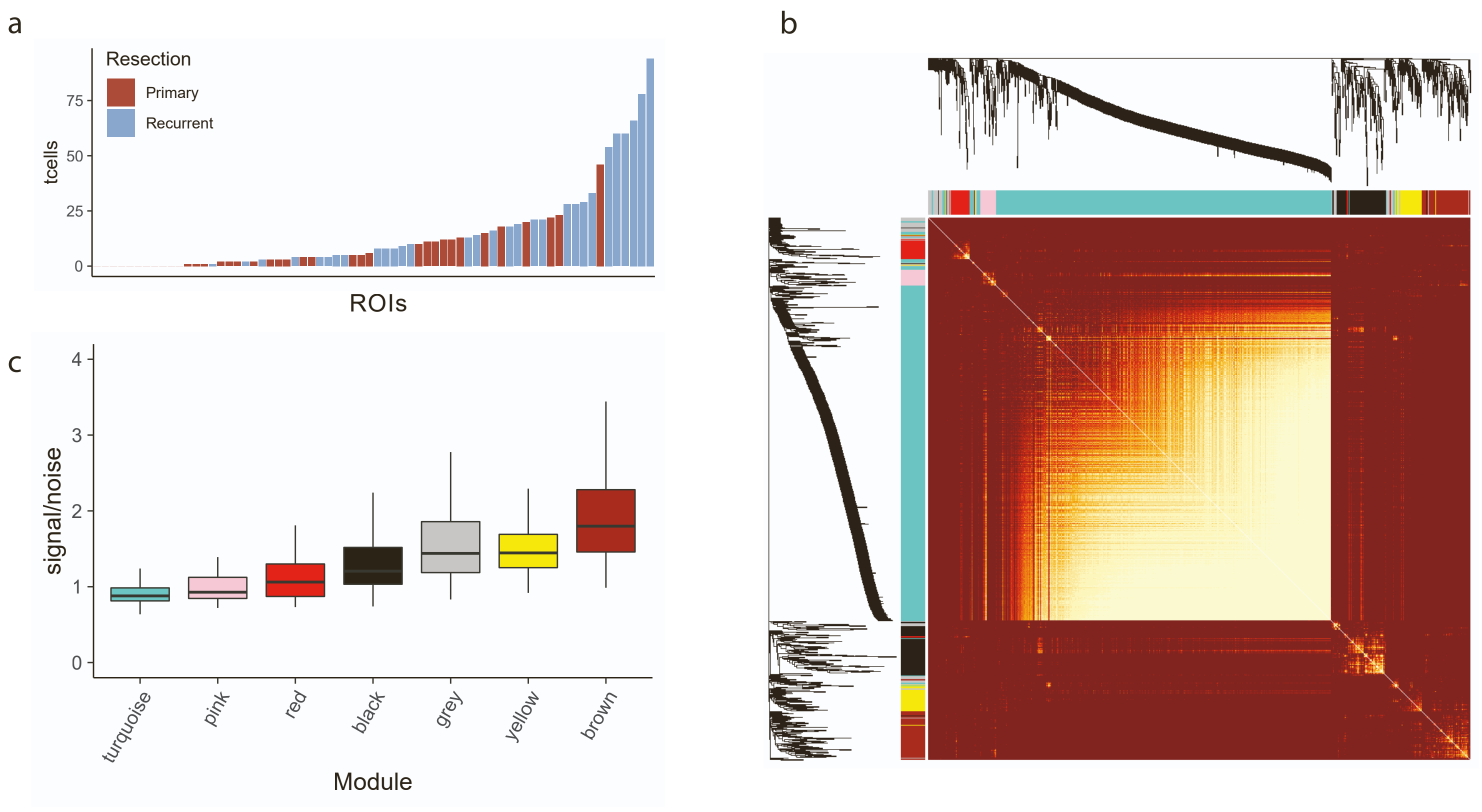

Supplementary Figure 3, related to Figure 3 | WGCNA analysis of Q3-normalized NanoString GeoMx DSP data shows an uneven distribution of modules likely due to a difference in signal-to-noise ratio. (a) Barplot of T cell count per ROI. (b), Heatmap showing the Topological Overlap Matrix (TOM) scale, which from red to yellow indicates the extent of relative overlap between genes. Dendrogram and gene module colours are shown along the top and side. (c), Boxplot of the signal to noise ratio distributions for the genes in each module. Rank is based on median signal-to-noise ratio. All boxplot boxes show the interquartile range (IQR) whiskers can extend to max 1.5 x IQR.
